# International Clinics

**Published:** 1893-02

**Authors:** 


INTERNATIONAL CLINICS: A Quarterly of Clinical Lectures on Medicine, Surgery, Gynecology, Pediatrics, Neurology, Dermatology, Laryngology, Ophthalmology, and Otology. By professors and lecturers in the leading medical colleges of the United States, Great Britain, and Canada. Edited by JOHN M. KEATING, M. D., Philadelphia, Consulting Physician for Diseases of Women to St. Agnes' Hospital, Philadelphia; editor Cyclopedia of the Diseases of Children. J. P. Crozer Griffith, M. D., Philadelphia, Clinical Professor of Diseases of Children in the University of Pennsylvania; Professor of Clinical Medicine in the Philadelphia Polyclinic. J. Mitchell Bruce, M. D., F. R. C. P., London, England, Physician and Lecturer on Therapeutics at the Charing Cross Hospital. David W. Finlay, M. D., F. R. C. P., Aberdeen, Scotland, Professor of the Practice of Medicine in the University of Aberdeen; Physician to and Lecturer on Clinical Medicine in the Aberdeen Royal Infirmary; Consulting Physician to the Royal Hospital for Diseases of the Chest, London. Second series. Volumes I., II., and III. 1892. Philadelphia: J. B. Lippincott Company.

These three volumes of the second series for 1892 continue the work as mapped out in the original plan. The distribution of the several subjects has been characterized by that good judgment for which the editors are famous.

In Volume I. we note, with special interest, Dr. McPhedran's lecture on Pernicious Anemia. This perplexing disease needs much elaboration and intricate study, and this author's contribution to the subject is valuable. Dr. Dercum's lecture on Paralysis Agitans is an able setting forth of this disease, and is well illustrated by five photographic reproductions of cases. In the department of pediatrics are a number of lectures that will prove of special interest to the practitioner in this important field of medicine. Dr. Rex gives a short lecture on the Treatment of Constipation in Children, which is excellent. In the surgical department, Dr. Vander Veer discourses upon Some Forms of Adominal Tumors in a most instructive manner, while Dr. Roberts treats of Several Forms of Joint Disease in his usual forcible style. Dr. Montgomery delivers an interesting lecture on Malignant Disease of the Uterus and Its Treatment, in which he gives an interesting experience with Kraske's method of extirpation.

In Volume II., Myxedema is discoursed upon by Dr. Finlay and Dr. Oliver, both of whom illustrate their articles, which furnishes an interesting and instructive addition to the lectures on this subject. In the department of neurology we find interesting and well illustrated lectures by Drs. Allen Starr, F. X. Dercum, and Hendrie Lloyd. Mr. Bland Sutton contributes a most interesting statement on the Axial Rotation of Abdominal Tumors. Inguinal Hernia and Herniotomy are treated of by Dr. Graham, of Washington, in an able manner, but his article deserves illustration. Uterine Hemorrhage is interestingly handled by Dr. Henry C. Coe, as is also the Treatment of Different Forms of Uterine Cancers by Dr. Ashby. Ophthalmology is ably represented by Dr. William Oliver Moore and Dr. McKenzie Davidson, while Laryngology and Rhinology are dealt with by Drs. John N. Mackenzie, Lefferts, and Casselbery. Dr. Ohmann-Dumesnil discourses upon Spontaneous Keloid, and presents a graphic illustration of a case. Dr. Charles H. Burnett closes the book on Some Common Forms of Disease of the Ear, which deserves attentive reading.

In Volume III., Dr. Alfred L. Loomis speaks of Suppurative Pleurisy, while Dr. Charles G. Stockton, of Buffalo, learnedly discourses upon Gastric Neuroses. An important lecture on Foreign Bodies in the Air Passages is given by Dr. Hector C. Cameron, of Glasgow; Dr. Forchheimer speaks ably upon Intestinal Catarrh, while Dr. Ernest Wende, of Buffalo, presents a most interesting and instructive lecture on Infantile Eczema, which is illustrated by a most remarkable case. Whenever Dr. Wende speaks, he teaches something, and no one can arise from the perusal of this lecture without feeling he has been communing with a master. De Garmo speaks on The Mechanical Treatment of Inguinal Hernia, which he illustrates by a number of appliances. Dr. Mann discourses upon the Removal of Diseased Tubes and Ovaries with Firm Adhesions, and Dr. Polk tells How to Preserve the Uterine Appendages by Operation. Berry Hart discourses upon Typical Abortion, and illustrates his paper with most excellent cuts. Dr. Mundé gives an interesting group of cases, which closes the gynecological lectures in this volume.

We have hastily run over these three volumes, making allusions here and there to some points of special interest, but have by no means been able to give a complete analysis of all the lectures in the three books. The space at our command would not permit us to do this, neither is it necessary in order to convey to the reader a proper understanding of the merits of this work. We commend it especially to teachers and clinicians, as well as to general practitioners, who are anxious to keep pace with the progress of medicine and surgery in its several departments.

